# Supplementary material for: Causal Relationship between Aging and Anorexia Nervosa: A White-Matter-Microstructure-Mediated Mendelian Randomization Analysis
Source: Biomedicines. 2024 Aug 16;12(8):1874. doi: 10.3390/biomedicines12081874 (PMC11351342; doi:10.3390/biomedicines12081874)
Supplement: Supplementary file 1 [file biomedicines-12-01874-s001.zip › Supplementary Figure/Figure S4/Singe SNP plot of MR analysis for TL to ubm-b-1516.pdf]

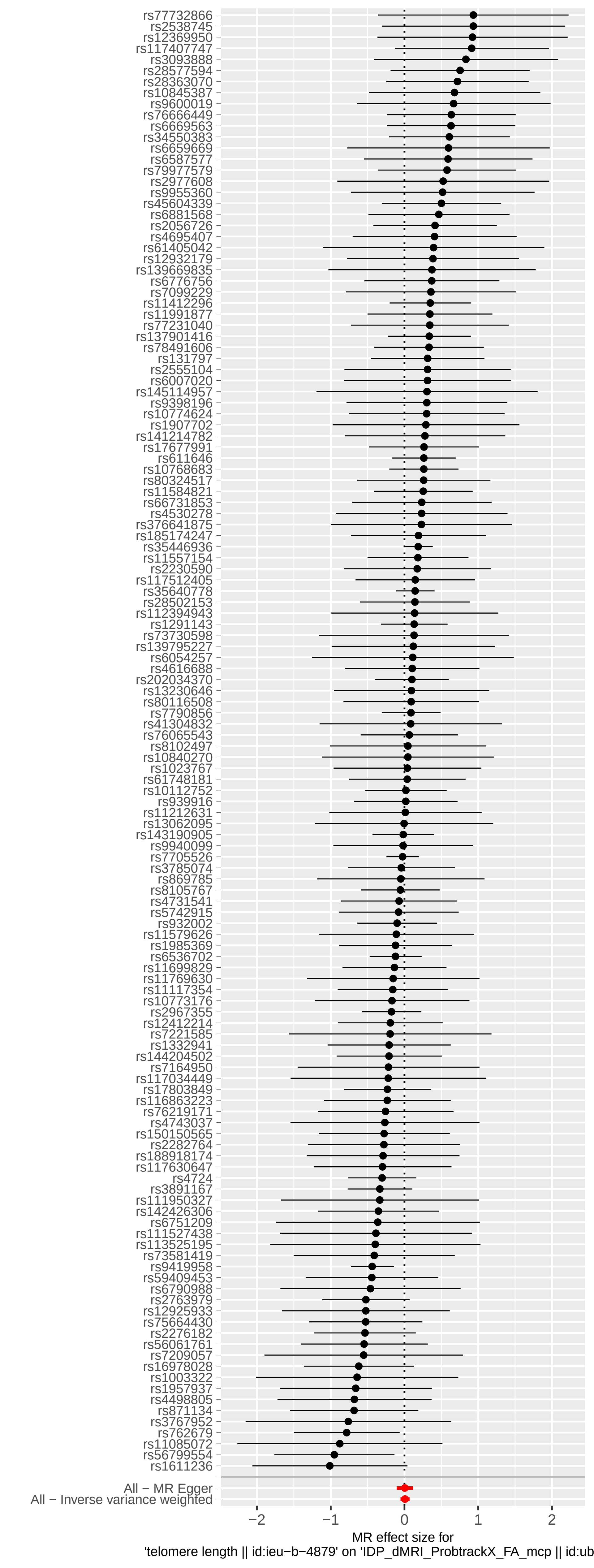
rs77732866
rs2538745
rs12369950
rs117407747
rs3093888
rs28577594
rs28363070
rs10845387
rs9600019
rs76666449
rs6669563
rs34550383
rs6659669
rs6587577
rs79977579
rs2977608
rs9955360
rs45604339
rs6881568
rs2056726
rs4695407
rs61405042
rs12932179
rs139669835
rs6776756
rs7099229
rs11412296
rs11991877
rs77231040
rs137901416
rs78491606
rs131797
rs2555104
rs6007020
rs145114957
rs9398196
rs10774624
rs1907702
rs141214782
rs17677991
rs611646
rs10768683
rs80324517
rs11584821
rs66731853
rs4530278
rs376641875
rs185174247
rs35446936
rs11557154
rs2230590
rs117512405
rs35640778
rs28502153
rs112394943
rs1291143
rs73730598
rs139795227
rs6054257
rs4616688
rs202034370
rs13230646
rs80116508
rs7790856
rs41304832
rs76065543
rs8102497
rs10840270
rs1023767
rs61748181
rs10112752
rs939916
rs11212631
rs13062095
rs143190905
rs9940099
rs7705526
rs3785074
rs869785
rs8105767
rs4731541
rs5742915
rs932002
rs11579626
rs1985369
rs6536702
rs11699829
rs11769630
rs11117354
rs10773176
rs2967355
rs12412214
rs7221585
rs1332941
rs144204502
rs7164950
rs117034449
rs17803849
rs116863223
rs76219171
rs4743037
rs150150565
rs2282764
rs188918174
rs117630647
rs4724
rs3891167
rs111950327
rs142426306
rs6751209
rs111527438
rs113525195
rs73581419
rs9419958
rs59409453
rs6790988
rs2763979
rs12925933
rs75664430
rs2276182
rs56061761
rs7209057
rs16978028
rs1003322
rs1957937
rs4498805
rs871134
rs3767952
rs762679
rs11085072
rs56799554
rs1611236
All − MR Egger
All − Inverse variance weighted
−2
−1
0
1
2
MR effect size for
'telomere length || id:ieu−b−4879' on 'IDP_dMRI_ProbtrackX_FA_mcp || id:ub
